# Intensive simulation versus control in the assessment of time to skill competency and confidence of medical students to assess and manage cardiovascular and respiratory conditions—a pseudo-randomised trial

**DOI:** 10.1186/s41077-016-0016-z

**Published:** 2016-05-30

**Authors:** Neil J. Cunningham, Robert O’Brien, Tracey Weiland, Julian van Dijk, Stuart Dilley

**Affiliations:** 1 St Vincent’s Clinical Education and Simulation Centre, St. Vincent’s Hospital, PO Box 2900, 41 Victoria Parade, Fitzroy, Melbourne, 3065 Australia; 2 Faculty of Medicine, Dentistry and Health Sciences, The University of Melbourne, Parkville, Australia; 3 Department of Medical Education, Melbourne Medical School, The University of Melbourne, Parkville, Australia; 4 Emergency Medicine Practice Innovation Centre, St Vincent’s Hospital, Melbourne, Australia

**Keywords:** Patient simulation, Education, medical, undergraduate, Clinical medicine, Educational assessment, Clinical competence, Education, curriculum

## Abstract

**Background:**

The Clinical Placement Enhancement Program (CPEP) is a simulation course for medical students learning the core topics of cardiovascular and respiratory medicine, incorporating patient safety and professionalism teaching and based on adult learning principles and proven educational theory. The aims of this study are to assess whether the CPEP delivered at the beginning of a clinical rotation would result in competency outcomes that are at least equivalent to those achieved through a standard 6-week programme and whether this programme would increase student confidence levels in assessing and managing patients with cardiovascular and respiratory conditions.

**Methods:**

This was a pseudo-randomised control trial between two groups of medical students from one clinical school. The intervention group participated in CPEP, a 4-day immersive simulation course, in the first week of their cardiac and respiratory medicine clinical rotation. The control group participants attended the normal programme of the 6-week cardiovascular and respiratory medicine clinical rotation. The programme and student competence was assessed using Observed Structured Clinical Examinations (OSCEs) and self-reported confidence surveys.

**Results:**

There was no significant difference in OSCE scoring between the intervention group (examined in week one of their clinical rotation following CPEP) and the control group (examined at the end of their full clinical rotation). Students exposed to CPEP started their clinical rotation with confidence levels similar to those reported by the control group at the end of their rotation. Confidence levels of CPEP students were higher at the end of the rotation compared to those of the control group.

**Conclusions:**

Based on OSCE results, immersion into a 4-day simulation-based teaching programme at the start of a clinical rotation resulted in skill competency levels that were equivalent to those obtained after a full clinical rotation of 6 weeks. CPEP improved students’ confidence levels in the assessment and management of patients presenting with cardiovascular and respiratory conditions. Simulation utilised in courses such as CPEP has the potential to enhance the overall learning experience in medical school clinical rotations.

**Electronic supplementary material:**

The online version of this article (doi:10.1186/s41077-016-0016-z) contains supplementary material, which is available to authorized users.

## Background

The traditional apprenticeship model of medical education is being challenged by multiple factors, including changes to medical school curricula, patient demographics, acute hospital workflow and senior clinician availability [1]. The breadth of medical student education often depends upon the number of ‘teaching cases’ available on the ward during a student’s term. The traditional ‘See one, do one, teach one [2]’ method occurs in an uncontrolled teaching arena and is associated with an unacceptable patient risk. New curriculum models and increasing student numbers are further stretching resources and resulting in decreased clinical time with patients. Acutely unwell patients and those with ‘clinical signs’ have decreased lengths of stay in hospital, resulting in potentially less exposure to these clinical signs for students and a subsequent reduction in valuable educational resources. Increasing demands upon senior clinicians are reducing the exposure of students to them. Senior clinicians rarely have the opportunity to observe a student moving through the following stages with a single patient’s history, examination, diagnosis and planning. Newly qualified doctors often struggle not with knowledge but with application—the approach to the undifferentiated sick patient, communication skills in the workplace and decision-making. Evaluation of new doctor performance is difficult and not standardised [1–4].

The Australian Medical Council noted that ‘(t)he challenge for all medical schools is to develop a curriculum which, while not neglecting the transmission of factual knowledge and practical skills, also stimulates enquiry, develops analytical ability and encourages the development of desirable professional attitudes in the students’ [5]. The Clinical Placement Enhancement Program (CPEP) course was designed to use a variety of interactive education modalities, including simulation, to optimise the delivery of two core medical school subjects—cardiovascular and respiratory medicine.

Simulation-based education is increasingly being used in healthcare including in the areas of patient safety and the development of procedural and clinical skills. Educational theory provides a basis for how simulation can be utilised in medical education. A simulator is a training device that artificially duplicates the conditions likely to be encountered in a particular situation. Simulation involves the operation of that training device over time [6]. In the setting of medical education, the simulator usually involves a mannequin (full size or part task training device) in an environment designed to mirror that which would be encountered by the student or doctor in real life. The simulation is run with the patient exhibiting symptoms and signs of a clinical condition, and the participant is required to work through the assessment, diagnosis and management of this condition. This is usually combined with a debrief, which is a guided reflection upon the behaviour during the simulation and an opportunity to discuss both strategies for improved performance and fill in any knowledge gaps that have been identified [7–11].

The two educational models adopted to underpin this project were Kolb’s learning model and Bloom’s taxonomy. Kolb’s model demonstrates how a cyclical learning model involving experience, reflection, conceptualisation and reapplication mirror the staged processes often incorporated into simulation-based education [12]. In the first stage, the simulation provides the ‘concrete experience’. The student interacts with the simulated patient and clinical staff (confederate acting as a nurse) in a realistic clinical environment. A debrief occurs in the second stage where the students reflect on their own experiences and those that they have observed. During the third stage, ‘forming abstract concepts’, the facilitator provides the students with concepts and assists in providing opportunity for conceptualization. The fourth stage, ‘testing in new situations, experimentation’, is a chance for the students to go back and repeat the simulation, with the unlimited opportunity to try things differently without the fear of harming patients. In single-task simulations, this takes the form of full repetition, with the expert facilitator guiding the feedback towards improved technical performance. In high-fidelity, immersive simulations, which have more variables and are logistically more difficult to replicate, the repetition often takes the form of recurring behavioural themes such as ‘calling for help’ or ‘allocation of roles’ [13, 14]. The twelve simulated clinical scenarios and subsequent debrief with senior clinicians in CPEP used this theory as a model for teaching.

Bloom’s taxonomy is viewed as a hierarchical schema of learning. Simulation-based education aims to fit into the educational model [15]. The accumulation of knowledge and facts occurs prior to the simulation. The expectation of a baseline level of knowledge is often assumed, but as noted above, the simulation offers an opportunity to identify knowledge gaps in participants. At the ‘application’ level, students use their knowledge in a new situation, having previously acquired this knowledge in an abstract setting. At the ‘analysis’ level, students apply critical thinking to a realistic clinical situation. At the ‘synthesis’ level, students have the opportunity to think creatively in a clinical environment.

The CPEP is a 4-day immersive simulation course, delivered in the first week of cardiac and respiratory medicine clinical rotations for final year medical students. The CPEP course was designed to integrate patient safety and professionalism teaching into a simulation course based around the core topics of cardiovascular and respiratory medicine, using adult learning principles and proven educational theory.

The aim of this study is to assess the effectiveness of the CPEP [16, 17]. Specifically, this paper addresses the following research questions:

1. Does the CPEP result in competency outcomes that are at least equivalent to those achieved through a standard 6-week teaching programme?
2. Does the CPEP lead to an improvement in students’ confidence in assessing and managing patients with cardiovascular and respiratory conditions?

## Methods

### Setting

This study was conducted at the St Vincent’s Clinical Education and Simulation Centre, St Vincent’s Hospital, Melbourne. Ethical approval was obtained through The University of Melbourne and St Vincent’s Hospital, Melbourne.

### Inclusion criteria

This includes The University of Melbourne, St Vincent’s Clinical School, final year medical students who were undertaking clinical rotations in cardiovascular and respiratory medicine at St Vincent’s Hospital. Prior to allocation, all students had received standard University of Melbourne education on cardiovascular and respiratory medicine as part of their undergraduate pre-clinical training.

### Recruitment, consent and pre-intervention data collection

Eligible students were invited to attend the CPEP course and the Observed Structured Clinical Examination (OSCE) testing days. Attendance was voluntary and written consent was obtained for all participants. Participants were requested to provide information regarding their knowledge, skills and attitudes in cardiovascular and respiratory medicine; self-rated proficiency in clinical skills; and basic demographic information (gender, age, cultural heritage, whether English is their first language, number of years speaking English).

### Participant allocation

**Fig. 1:**
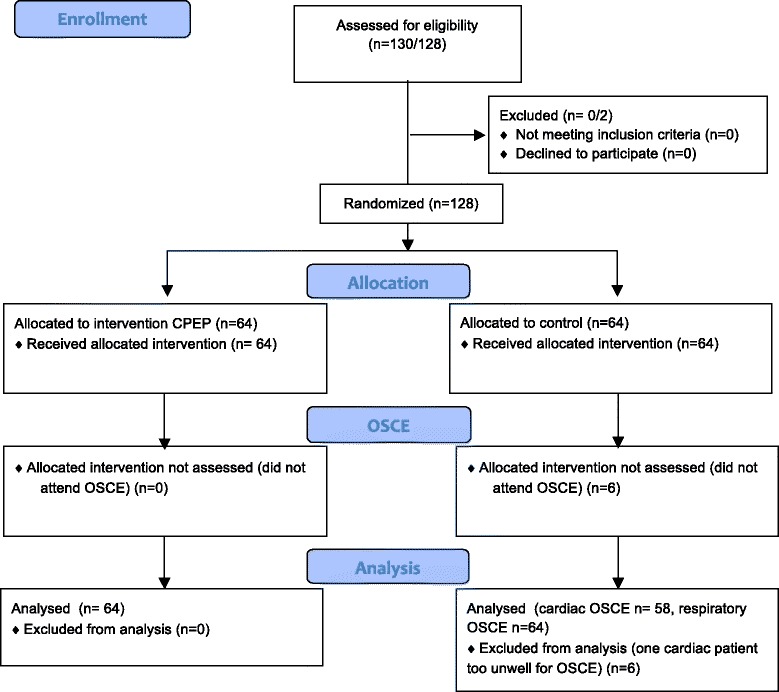
Randomisation and participant allocation. *CPEP* Clinical Placement Enhancement Program, *OSCE* Observed Structured Clinical Examination

Participants were pseudo-randomised to the project by St Vincent’s Hospital, The University of Melbourne Clinical School (Fig. 1) [18]. All participants were allocated a random identifier number to label all collectable data sheets during the study. The University of Melbourne Clinical School decided determination of control and intervention group allocation, and the course dates were set to coincide with the commencement of clinical rotations. Six CPEP courses were conducted over a 2-year period. Participants and rating clinicians were both blinded to group allocation.

### Procedure

The control group participants attended the normal programme of the 6–8-week cardiovascular and respiratory medicine clinical rotation.

The intervention group participated in CPEP, a 4-day immersive simulation course, in the first week of their cardiac and respiratory medicine clinical rotation. They then completed the remaining 5–7 weeks of the cardiovascular and respiratory rotation.

### CPEP course

The CPEP was a 4-day course designed to provide students with intensive education at the start of their cardiovascular and respiratory clinical placements (Additional file 1). It used a combination of interactive teaching methods to guide the students in essential areas of key knowledge, skills and attitudes. It encouraged students to reflect upon their current level of performance in patient assessment and management and to self-identify areas of deficit. The course was designed to augment and not replace the current cardiovascular and respiratory curriculum and integrate patient safety, communication skills, professionalism and a teaching model of Kolb throughout.

The interactive teaching methods included:

- Four facilitated discussions covering the core areas of cardiovascular and respiratory physiology, ECG and chest X-Ray analysis.
- Four simulation-based “bedside tutorials” using the high-fidelity mannequin for real-time demonstration and practice of clinical examination (cardiovascular and respiratory), vasoactive pharmacology and iPod-generated pathological heart sounds.
- Twelve simulated scenarios using the high-fidelity mannequin giving the students the opportunity to group problem-solve clinical scenarios in a highly stimulating and clinically realistic environment. This allowed them to practise confronting unfamiliar problems and complex concepts, while expressing orally the process and result of team problem solving. These scenarios were debriefed by senior medical staff and simulation experts who reviewed the scenario and directed a discussion around the key clinical issues. These educators were not involved in the later assessments.

### Control

The normal programme of the 6–8-week cardiovascular and respiratory medicine clinical rotations consisted of a combination of formal and informal teaching. The formal component included lectures and attendance at clinical meetings. The informal component included history taking and examination of patients, case presentations to registrar/consultant and observation of clinical staff in their day to day work. This observed clinical work would involve communicating with and examining patients, ordering investigations and performing procedures. There was no simulation-based education as part of the normal clinical rotations.

### Outcomes—time to develop competencies

The primary outcome was the participant’s score on a subsequent OSCE exam—this was to analyse whether the intervention group could perform competently after CPEP in comparison to the control group who were being assessed at the end of their full rotations.

The OSCE score was taken at one time-point only for each participant; baseline measures were not taken.

The OSCE was chosen as an objective-testing tool as it was the current standardised examination for undergraduate cardiovascular and respiratory medicine used by The University of Melbourne. OSCEs are currently conducted around the world and provide an opportunity to be rated according to a global rating scale against a range of competencies. OSCEs are set for a range of knowledge areas and can be conducted in the same way in a variety of environments [16, 19, 20].

All participants attended an examination day, participating in the same two cardiovascular and respiratory OSCE stations. These OSCE stations consisted of examining and presenting an assessment of actual patients, as would occur during University of Melbourne examinations. The OSCEs were scored based on a marking criteria scoring key used by The University of Melbourne, out of possible total of 30 marks. OSCE data was collected at one point for each intervention and control group. The CPEP group attended OSCE data collection on the day after the course had finished; the control group attended in the week following their 6-week cardiovascular and respiratory rotation. All OSCE examiners were experienced, having previously examined OSCE stations for The University of Melbourne Medical School using the same scoring key. OSCE examiners were blinded to group allocation. The same examiners were not available for all the OSCE examinations.

### Outcomes—confidence rating

Secondary outcome measures were obtained from participant surveys—baseline, post-course, and end of rotation. The surveys were designed to record self-rated confidence in knowledge, skills and management tasks for 17 clinical skills relevant to cardiovascular and respiratory medicine (Additional file 2). A five-point multi-category rating scale of confidence was used with descriptors (never confident: I am challenged or threatened by this topic or skill; rarely confident: I am well outside my comfort zone; sometimes confident: I am sometimes confident with this topic or skill but could improve; usually confident: I am comfortable with this topic or skill; always confident: I am expert at this topic or skill). Basic demographic information was also obtained to assist in the assessment of confounding factors for their clinical skill development.

The baseline survey was given to all participants on day 1 of their clinical rotations (day 1 of CPEP, prior to commencement of the teaching programme).

The post-course survey was given to the intervention group immediately following completion of the course.

The end of rotation survey was given to all participants at the end of their rotation. An analysis of these follow-up surveys, using the same confidence-rating scales, was conducted to examine the self-assessment scoring of the control and intervention groups participants.

### Sample size estimation

Based on the existing data for OSCE performance, it was anticipated that the control group would have a mean OSCE score of 70.0 ± 23.1 (SD). In order to achieve a mean difference of 12 points between the simulation-exposed group and controls, a sample size of 59 per group was required with alpha set at 0.05 (two-tailed) and power set at 80 %. Assuming a dropout rate of approximately 10 %, a sample size of 65 per group was planned.

### Statistical analyses

Demographic data were collected on a standard pro forma directly from the participants. OSCE scores were collected following each examination session. Baseline, post-course and follow-up survey data were collected directly from the participants. Data were analysed using the Statistical Package for the Social Sciences (SPSS; version 15.0, IBM). Descriptives (number, percentage) were calculated for demographics and OSCE data (median, interquartile range (IQR)). OSCE total scores for the intervention and control groups were not normally distributed and were therefore compared using the Mann-Whitney *U* test. OSCE subcomponents for intervention and control groups were analysed using Fisher’s exact test for categorical data.

For inferential analyses, confidence data were collapsed from five response categories (‘never confident’, ‘rarely confident’, ‘sometimes confident’, ‘usually confident’ and ‘always confident’) to two: ‘never/rarely/sometimes confident’ and ‘usually/always confident’. Confidence data for intervention and control groups were compared for baseline and for post-test using Fisher’s exact test. Two-tailed tests of significance were used in all instances and alpha was set at 0.05.

## Results

During the study period, all students (130) attended for their cardiovascular/respiratory rotations (64 CPEP, 66 control). Data assessing skill competency based upon OSCE testing were available for 122 out of 130 students, 64/64 from the intervention group and 58/66 from the control group. Some data sets were incomplete: eight students from the control group did not attend OSCE; one group of five students (CPEP group) was unable to complete the CVS OSCE as the allocated patient was unwell and unable to attend the examination (117/122 for CVS OSCE, 122/122 for respiratory OSCE); one person did not complete the demographic component of their survey (121/122).

### Demographics

The students were aged between 20 and 36 (mean 23.55, standard deviation 3.078) and included direct school leavers (47.1 %), graduates (23.1 %) and international students (31.4 %). There were 53 males (43.8 %) and 68 females (56.2 %). Respondents identified 22 different cultural backgrounds, the majority describing themselves as either Chinese (28.9 %) or Australian (26.4 %). English as a second language was spoken by 47 (38.8 %) students, and of these, some had only been speaking English for 2 years.

### Competency outcomes—OSCE data

There were no significant differences in OSCE scores between the intervention group assessed at the end of CPEP and the control group assessed at the end of rotation.

The median respiratory OSCE score for the CPEP group (*N* = 64) was 25.0 (IQR 23.0–27.0) compared to 26.0 (IQR 22.5–28.5) for the control group (*N* = 58) (*P* = 0.08). The median cardiovascular OSCE score for the CPEP group (*N* = 58) was 26.0 (IQR 23.7–27.0) compared to 25.7 (IQR 23.7–27.0) for the control group (*N* = 58) (*P* = 0.176).

One of the actual patients (cardiac) was too unwell to participate in the OSCE.

### Confidence rating—survey data

**Fig. 2:**
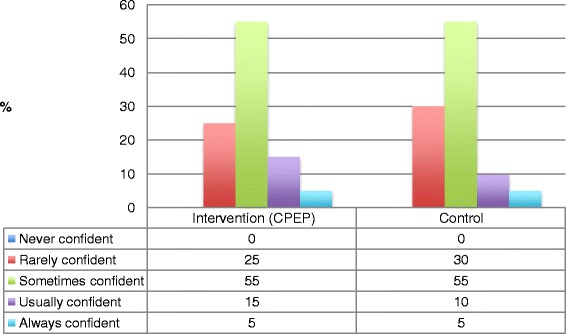
Baseline ‘commencement of rotation’ survey comparison data—‘Do you feel confident with cardiovascular and respiratory medicine?’ *CPEP* Clinical Placement Enhancement Program

**Fig. 3:**
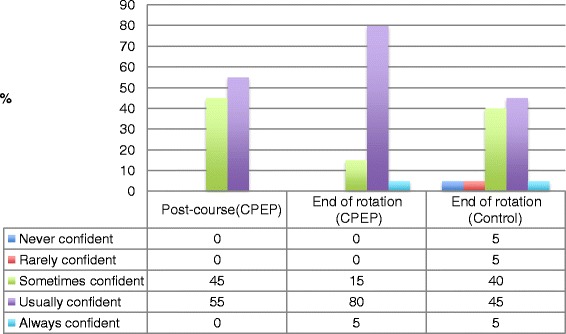
Survey comparison data—‘Do you feel confident with cardiovascular and respiratory medicine?” *CPEP* Clinical Placement Enhancement Program

**Fig. 4:**
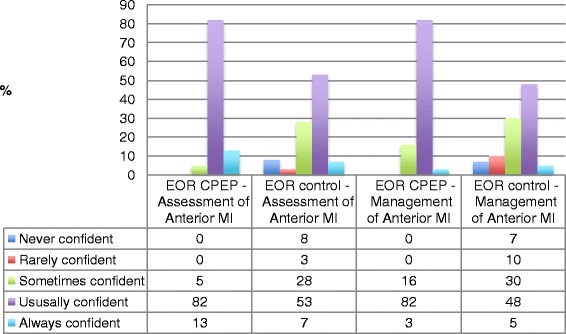
‘End of rotation’ (EOR) survey data for CPEP and control groups—self-rated confidence in assessment and management of AMI. *EOR* end of rotation, *CPEP* Clinical Placement Enhancement Program, *MI* myocardial infarction

Data relating to students’ self-reported confidence levels are presented in Figs. 2, 3 and 4.

Students in both groups (CPEP and control) self-reported similar levels of confidence in managing cardiovascular and respiratory conditions at prior to commencement of CPEP and clinical placements (Fig. 2).

At the end of the 4-day CPEP intervention, CPEP students reported confidence levels that were similar to those reported by the control group at the end of their 6-week placement (Fig. 3). At the end of their 6-week placement, 84.2 % of CPEP students reported that they were ‘usually’ or ‘always’ confident in managing cardiorespiratory conditions compared with 48.2 % of those in the control group (Fig. 3).

Self-reported confidence in the assessment and management of acute myocardial infarction are shown in Fig. 4.

The full results from the end of rotation surveys are available as online supplemental material in Additional file 3.

## Discussion

The aim of this study was to assess the benefit of the CPEP with respect to medical student competence and confidence.

### Competency outcomes—OSCE data

The primary objective of this study was to assess whether an intensive 4-day simulation course would result in competency outcomes that were comparable to those achieved by a standard 6-week clinical rotation in cardiovascular and respiratory medicine. This was not with the intention of replacing the clinical rotation but to enhance the student’s experience of the rotation by providing them with a strong clinical platform. This comparison used the current assessable standard, the OSCE. The data collected from the OSCEs demonstrated that there was no statistical difference between the intervention group after the first week and the control group OSCE scores at the end of 6 weeks.

This data suggests that at the completion of 4 days of intensive simulation-based education, the intervention group were able to attain the same level of knowledge and skills that the control group had achieved over a 6–8-week period.

### Confidence rating—survey data

The secondary objective was to assess student’s confidence in managing cardiovascular and respiratory problems. Self-reported confidence survey results provided a comparison of the ‘post-course’ (intervention) and the ‘end of rotation’ (control) groups. This demonstrated that students exposed to CPEP went into the remaining 5 weeks of their rotation with confidence levels that were similar to levels reported by the control group when they were at the end of their rotation*.* End of rotation survey confidence scores for the CPEP group showed nearly twice as many rating themselves as ‘usually, always confident’ compared to the control group. This displayed a further increase in confidence from the immediate post-course results*.*

Front-loading education for the rotation with the intervention group provided the students with an opportunity to build upon their knowledge, skills, and confidence for the remaining 5 weeks. In contrast, there is a more gradual development in the control group and they did not necessarily have the base to build their practical experiences upon. This may have wider curriculum applications with the option to concentrate key knowledge and skill components in the early phase of clinical rotations to optimise efficacy. This may be applicable with or without the use of simulation-based learning and is not limited to medical undergraduate teaching.

The CPEP course had originally been designed to clinically prepare the students for clinical practice, rather than how to pass an OSCE. In fact, the students had only two modules out of a total 16 that were directly related to clinical examination. These modules were presented as interactive sessions (‘how to learn how to do a cardiac/respiratory clinical examination’) and were designed to place the actual clinical relevance of an examination into a real-life context. The same skills and knowledge in examination were provided to the control group at various points in their rotation. Most clinicians and educators would expect students to emerge from a cardiovascular/respiratory term with some core fundamental knowledge and skills in place.

At the conclusion of the CPEP course, all of the intervention students went into their rotation rating themselves as either ‘sometimes or usually confident’. This effect continued to the end of rotation survey with all intervention responders rating themselves as ‘sometimes, usually or always confident’ at this point. Although it is possible that CPEP group of students developed falsely elevated confidence levels through their involvement in the course, the OSCE results suggested that the CPEP students were at an acceptable ‘end of rotation’ level at that point. If we compare this to the control group, it is concerning that there are a number of students who finish a key rotation with no confidence in their ability to practise as a doctor in this area. In fact, there were consistently higher responses that scored ‘rarely or never confident’ in the control group throughout the end of rotation subgroup analysis when compared to intervention group. Anecdotal comments from the participants suggest that some of the intervention group used the CPEP course to become aware early in their rotation about what they did and did not know, closing the gap between perceived and actual knowledge.

There are aspects of the clinical rotation that are difficult to replicate in simulation and are equally hard to measure. These include the real-time observation of clinical practice throughout days or weeks and the social and communication challenges that exposure to real patients provides. Allowing students to actively manage undifferentiated or acutely unwell patients is problematic due to the expectation that more senior staff would take over and the variability of exposure to these types of patients. Take the example of an acute myocardial infarction (AMI)—a heart attack, the leading cause of death in Australia [21]. The expectation would be that a student would learn how to assess and manage this common and life-threatening condition during their undergraduate clinical placement on a cardiology ward. A proportion of the control group finished their rotations being ‘rarely or never confident’ in their ability to either assess (11 % compared to 0 % intervention group) or manage (17 % compared to 0 % intervention group) an AMI (Fig. 4). CPEP did not specifically target the assessment and management of AMI; there were two simulation scenarios and an ECG facilitated discussion during the course.

Despite the prevalence of AMI, it has become increasingly common for medical students to progress through a cardiology term and not be exposed to this condition. Due to the different phases occurring along a patient’s journey from initial diagnosis and treatment (general practice, ambulance or emergency department) to definitive management (thrombolysis or primary coronary angioplasty) and ongoing care on the ward a student could easily go through their whole cardiology term without seeing an undifferentiated patient presenting with an AMI. On a cardiology ward, they are more likely to see an AMI patient after someone else more senior has diagnosed and treated them. The students therefore miss the learning process of taking a history in real time from a patient with chest pain, seeing where all the interventions (aspirin, oxygen, heparin, angiography, etc.) fit in, and communicating with a sick patient and other staff simultaneously. Simulation in a course such as CPEP has the ability to provide medical students with exposure to common conditions presenting as undifferentiated cardinal symptoms (chest pain and breathlessness) that they can attempt to assess and manage. This type of learning prepares the students to become clinically astute junior doctors who have been taught, via senior clinician modeling and feedback, how to approach sick patients with an unknown diagnosis, rather than a defined clinical condition—practicing being a doctor not a medical student. A clinically orientated simulation course provides the student with the opportunity to ask questions of and receive feedback from clinical experts. Time is quarantined for both the educator and the student without the need to compete with other students for patient or clinician time. Learning goals were not limited to specific clinical skills and the students received feedback on teamwork, communication and other non-technical/ professional skills. The key component of having expert feedback cannot be underestimated as previous problem-based curriculum changes may result in improved communication skills but reduced understanding of disease [2, 3].

Simulation fits the criteria of a modern, integrated educational approach designed to meet the challenge of finding ‘other educational strategies that promote student-centred rather than teacher-centred learning, promote active student enquiry, stimulate analytical and knowledge organisation skills, and foster lifelong learning skills’. [5]

### Limitations

The authors identified several limitations to this study that may have impacted upon the results.

The OSCE data could suggest that the behaviour marking structure for the assessment is compromised as it assesses a student’s ability to undertake pattern recognition with little if any weighting in assessment being given to the student’s ability to understand why they are performing a task. The OSCE did not measure whether the intervention and control groups had similar levels of understanding and context of why the physical examinations were undertaken. Due to logistical reasons, not all of the participants were assessed by the same examiners which may have lead to some score variation.

A pseudo-randomisation strategy was used due to a number of logistical constraints. Access to the medical students was controlled and organised by The University of Melbourne’s Clinical School at St Vincent’s Hospital. As a result, the research team at St Vincent’s Clinical Education and Simulation Centre did not make the rotation group allocations.

We were advised that student allocation to clinical groups follows a process to maintain group diversity. Clinical groups are 7 to 8 persons in size, gender balanced and with the group population representing three criteria—direct school leavers, graduate students and international students. Groups are also gender balanced.

There are two issues that may have affected the control groups in this study. Not all of the clinical rotations were exactly 6 weeks, and therefore, control group OSCE testing did not always occur exactly at the 6-week mark. Where logistically possible, control groups were allocated OSCE testing times with the intervention group, on the Friday immediately following the 4-day CPEP. In order to achieve this, some control groups attended combined testing at weeks 5 or 7 and 8.

Two out of the six control groups involved in the study attended the end of rotation OSCE stations as an unmixed group (no intervention group participants). To minimise the impact of this potential bias for the OSCE examiners, all examiners were external to the CPEP and blinded as to what group is being examined. The examiners only dealt with a participant by number at the OSCE station they had been allocated to run. No teaching staff from the CPEP were in any way involved in the running of the OSCE examination stations.

In addition, due to limitations in access to resources and access to students the research group felt that the OSCE results may have provided greater assistance if there had been testing pre-rotation/pre-intervention, at the completion of the intervention/first week of rotation and at the completion of the rotational block. These three points for both the intervention and control group may have provided a further understanding as to whether there were any differences in rate of skill acquisition and retention as a result of the program. Although the data shows that the CPEP group after 1 week obtained similar OSCE scores to the control group who had undergone a full rotation, it is not possible to say that the time to skill competency was reduced, as there was no comparison control group OSCE taken at 1 week into the rotation.

Strengths of this study include the validity of groups for comparison, the collection of quantitative as well as qualitative data and the application of the standard assessment tool for this target group—the OSCE.

## Conclusions

Introduction of the CPEP teaching programme provided the same level of assessable skill competency in medical students after 4 days as those who had undergone a full clinical rotation of 6 weeks. CPEP improved students’ confidence levels in the assessment and management of patients presenting with cardiovascular and respiratory conditions. CPEP gave the students clinical relevance, exposure to important knowledge gaps and the basic technical language tools to start learning cardiovascular and respiratory medicine on their rotations. Simulation-based education utilised in courses such as CPEP has the potential to enhance the overall learning experience in medical school clinical rotations.

### Research questions

- Does the introduction of the 4-day CPEP teaching programme at the beginning of a clinical rotation lead to acquisition of skill competency as measured by OSCE that is equivalent to competence obtained from a 6-week clinical placement?
- Does the introduction of the CPEP teaching programme at the beginning of a clinical rotation lead to improvements in students’ confidence levels in assessing and managing patients with cardiovascular and respiratory conditions?

### Main messages

- A 4-day CPEP course at the beginning of a medical student clinical rotation resulted in skill competency levels equivalent to those obtained from a 6-week clinical placement.
- A 4-day CPEP course improved students’ confidence levels in the assessment and management of patients presenting with cardiovascular and respiratory conditions.
- Simulation has the potential to enhance the overall learning experience in medical school clinical rotations.

## Additional files

Additional file 1: CPEP timetable. CVS—cardiovascular system, MI—myocardial infarction, GTN—glyceryl trinitrate, ECG—electrocardiogram, CXR—chest X-ray, SVT—supra-ventricular tachycardia, VT—ventricular tachycardia, SOB—short of breath, COPD—chronic obstructive pulmonary disease, CP—chest pain, PE—pulmonary embolism, APO—acute pulmonary oedema, OSCE—Objective Structured Clinical Examination.

Additional file 2: Self-rated confidence in knowledge, skills and management tasks. CVS—cardiovascular system, ECG—electrocardiogram.

Additional file 3: “End of rotation” survey data, control and intervention (CPEP).
