# Supplementary material for: Intensive simulation versus control in the assessment of time to skill competency and confidence of medical students to assess and manage cardiovascular and respiratory conditions—a pseudo-randomised trial
Source: Adv Simul (Lond). 2016 May 30;1:15. doi: 10.1186/s41077-016-0016-z (PMC5806281; doi:10.1186/s41077-016-0016-z)
Supplement: Supplementary file 1 — CPEP timetable. CVS—cardiovascular system, MI—myocardial infarction, GTN—glyceryl trinitrate, ECG—electrocardiogram, CXR—chest X-ray, SVT—supra-ventricular tachycardia, VT—ventricular tachycardia, SOB—short of breath, COPD—chronic obstructive pulmonary disease, CP—chest pain, PE—pulmonary embolism, APO—acute pulmonary oedema, OSCE—Objective Structured Clinical Examination. [file 41077_2016_16_MOESM1_ESM.pdf]

	Monday	Tuesday	Wednesday	Thursday	Friday
**0830 – 1000**	***1 – Introduction***<br>Intro to simulation, aims of course, familiarisation	***5 – Vasoactive pharmacology***<br>Simulation-based bedside tutorial<br>Inotropes, Beta-blockers, GTN, anti-arrhythmics	***9 – Respiratory exam***<br>Simulation-based bedside tutorial<br>Normal examination - mannequin and each other	***13 - Simulation***<br>SOB - young patient, asthma<br><br>SOB - old patient, COPD	***17 – OSCE CVS***
**Morning Tea**					
**1020 – 1200**	***2 – CVS examination***<br>Simulation-based bedside tutorial<br>Normal examination - mannequin and each other	***6 – ECG tutorial***<br>Facilitated discussion,<br>ECG case studies	***10 – Respiratory physiology***<br>Facilitated discussion	***14– Simulation***<br>SOB – young patient, aspiration pneumonia, drug abuse<br><br>SOB – old patient, from NH, CVA	***18 - OSCE CVS***
**Lunch**					
**1300 – 1445**	***3 - CVS physiology***<br>Facilitated discussion including cardiac cycle and valves	***7 – Heart sounds***<br>Simulation-based bedside tutorial<br>iPod/ examination cases	***11 - Simulation***<br>Bradycardia - complete heart block<br><br>Bradycardia – Beta blocker overdose	***15 – Simulation***<br>CP and SOB – PE<br><br>CP and SOB - APO	***19 - OSCE Respiratory***
**Afternoon Tea**					
**1500 - 1630**	***4 – Simulation***<br>Anterior MI - pump problem<br><br>Inferior MI - preload problem	***8 – CXR***<br>Facilitated discussion normal and pathology cases	***12 - Simulation***<br>Tachycardia – SVT<br><br>Tachycardia - VT	***16 – Round up.***<br>Post-course Evaluation	***20 - OSCE Respiratory***
