# Supplementary material for: Intensive simulation versus control in the assessment of time to skill competency and confidence of medical students to assess and manage cardiovascular and respiratory conditions—a pseudo-randomised trial
Source: Adv Simul (Lond). 2016 May 30;1:15. doi: 10.1186/s41077-016-0016-z (PMC5806281; doi:10.1186/s41077-016-0016-z)
Supplement: Supplementary file 2 — Self-rated confidence in knowledge, skills and management tasks. CVS—cardiovascular system, ECG—electrocardiogram. [file 41077_2016_16_MOESM2_ESM.pdf]

**CONFIDENCE**<br><br>**Do you feel confident with these topics and skills?**	**1**<br>**Never confident**<br><br>I am challenged or threatened by this topic or skill	**2**<br>**Rarely confident**<br><br>I am well outside my comfort zone	**3**<br>**Sometimes confident**<br><br>I am sometimes confident with this topic or skill but could improve	**4**<br>**Usually confident**<br><br>I am comfortable with this topic or skill	**5**<br>**Always confident**<br><br>I am expert at this topic or skill

	**Confidence in performing this task or skill**
	1. Never Confident<br>2. Rarely<br>3. Sometimes<br>4. Usually<br>5. Always Confident

	**1**	**2**	**3**	**4**	**5**
Standard CVS examination techniques					
Knowledge of CVS Physiology					
Assessment of Anterior Myocardial Infarction					
Management of Anterior Myocardial Infarction					
Assessment of Inferior Myocardial Infarction					
Management of Inferior Myocardial Infarction					
Knowledge and effects of drugs on the CVS					
Eliciting clinical signs in CVS examinations					
Understanding what an ECG is					
Understanding how to perform an ECG					
Interpreting an ECG					
Knowledge of Respiratory Physiology					
Knowledge and effects of drugs on Respiratory System					
Interpreting normal Chest X-Rays					
Interpreting abnormal Chest X -Rays					
Assessing patients with both CVS and Respiratory complications					
Managing patients with both CVS and Respiratory complications					
