# Supplementary material for: Intensive simulation versus control in the assessment of time to skill competency and confidence of medical students to assess and manage cardiovascular and respiratory conditions—a pseudo-randomised trial
Source: Adv Simul (Lond). 2016 May 30;1:15. doi: 10.1186/s41077-016-0016-z (PMC5806281; doi:10.1186/s41077-016-0016-z)
Supplement: Supplementary file 3 — “End of rotation” survey data, control and intervention (CPEP). [file 41077_2016_16_MOESM3_ESM.pdf]

		Never Confident % (n)	Rarely % (n)	Sometimes % (n)	Usually % (n)	Always Confident % (n)	Total (n)
Standard CVS (cardiovascular system) examination techniques	control	0.00% (0)	1.67% (1)	21.67% (13)	65.00% (39)	11.67% (7)	60
	CPEP	0.00% (0)	0.00% (0)	0.00% (0)	71.05% (27)	28.95% (11)	38
Knowledge of CVS Physiology	control	3.39% (2)	1.69% (1)	38.98% (23)	55.93% (33)	0.00% (0)	59
	CPEP	0.00% (0)	0.00% (0)	10.53% (4)	78.95% (30)	10.53% (4)	38
Assessment of Anterior Myocardial Infarction	control	8.33% (5)	3.33% (2)	28.33% (17)	53.33% (32)	6.67% (4)	60
	CPEP	0.00% (0)	0.00% (0)	5.26% (2)	81.58% (31)	13.16% (5)	38
Management of Anterior Myocardial Infarction	control	6.67% (4)	10.00% (6)	30.00% (18)	48.33% (29)	5.00% (3)	60
	CPEP	0.00% (0)	0.00% (0)	23.68% (9)	73.68% (28)	2.63% (1)	38
Assessment of Inferior Myocardial Infarction	control	6.67% (4)	8.33% (5)	28.33% (17)	48.33% (29)	8.33% (5)	60
	CPEP	0.00% (0)	0.00% (0)	5.26% (2)	81.58% (31)	13.16% (5)	38
Management of Inferior Myocardial Infarction	control	6.67% (4)	10.00% (6)	35.00% (21)	43.33% (26)	5.00% (3)	60
	CPEP	0.00% (0)	0.00% (0)	23.68% (9)	73.68% (28)	2.63% (1)	38
Knowledge and effects of drugs on CVS	control	5.08% (3)	3.39% (2)	37.29% (22)	54.24% (32)	0.00% (0)	59
	CPEP	0.00% (0)	2.63% (1)	23.68% (9)	71.05% (27)	2.63% (1)	38
Eliciting clinical signs in CVS exams	control	5.00% (3)	0.00% (0)	38.33% (23)	50.00% (30)	6.67% (4)	60
	CPEP	0.00% (0)	0.00% (0)	13.16% (5)	76.32% (29)	10.53% (4)	38
Understanding what an ECG (electrocardiogram) is	control	3.33% (2)	1.67% (1)	28.33% (17)	53.33% (32)	13.33% (8)	60
	CPEP	0.00% (0)	0.00% (0)	0.00% (0)	76.32% (29)	23.68% (9)	38
Understanding how an ECG operates	control	3.33% (2)	1.67% (1)	28.33% (17)	60.00% (36)	6.67% (4)	60
	CPEP	0.00% (0)	0.00% (0)	2.63% (1)	76.32% (29)	21.05% (8)	38
Interpreting an ECG	control	1.67% (1)	6.67% (4)	45.00% (27)	41.67% (25)	5.00% (3)	60
	CPEP	0.00% (0)	0.00% (0)	23.68% (9)	73.68% (28)	2.63% (1)	38
Knowledge of Respiratory Physiology	control	5.00% (3)	5.00% (3)	40.00% (24)	50.00% (30)	0.00% (0)	60
	CPEP	0.00% (0)	0.00% (0)	21.05% (8)	76.32% (29)	2.63% (1)	38
Knowledge and effects of drugs on Respiratory System	control	5.08% (3)	8.47% (5)	50.85% (30)	32.20% (19)	3.39% (2)	59
	CPEP	0.00% (0)	5.26% (2)	13.16% (5)	71.05% (27)	10.53% (4)	38
Interpreting normal Chest X-Rays	control	5.08% (3)	3.39% (2)	52.54% (31)	33.90% (20)	5.08% (3)	59
	CPEP	0.00% (0)	2.63% (1)	10.53% (4)	78.95% (30)	7.89% (3)	38
Interpreting abnormal Chest X-Rays	control	5.08% (3)	8.47% (5)	52.54% (31)	30.51% (18)	3.39% (2)	59
	CPEP	0.00% (0)	2.63% (1)	39.47% (15)	55.26% (21)	2.63% (1)	38
Assessing patients with both CVS and Respiratory complications	control	1.67% (1)	5.00% (3)	51.67% (31)	40.00% (24)	1.67% (1)	60
	CPEP	0.00% (0)	0.00% (0)	28.95% (11)	68.42% (26)	2.63% (1)	38
Managing patients with both CVS and Respiratory complications	control	1.82% (1)	7.27% (4)	54.55% (30)	34.55% (19)	1.82% (1)	55
	CPEP	0.00% (0)	7.89% (3)	36.84% (14)	50.00% (19)	5.26% (2)	38
